# Attitude of medical students towards Early Clinical Exposure in learning endocrine physiology

**DOI:** 10.1186/1472-6920-7-30

**Published:** 2007-09-05

**Authors:** Solomon Sathishkumar, Nihal Thomas, Elizabeth Tharion, Nithya Neelakantan, Rashmi Vyas

**Affiliations:** 1 Department of Physiology, Christian Medical College, Vellore, India; 2 Department of Endocrinology, Christian Medical College, Vellore, India; 3 Department of Biostatistics, Christian Medical College, Vellore, India

## Abstract

**Background:**

Different teaching-learning methods have been used in teaching endocrine physiology for the medical students, so as to increase their interest and enhance their learning. This paper describes the pros and cons of the various approaches used to reinforce didactic instruction in endocrine physiology and goes on to describe the value of adding an Early Clinical Exposure program (ECE) to didactic instruction in endocrine physiology, as well as student reactions to it as an alternative approach.

**Discussion:**

Various methods have been used to reinforce didactic instruction in endocrine physiology such as case-stimulated learning, problem-based learning, patient-centred learning and multiple-format sessions. We devised a teaching-learning intervention in endocrine physiology, which comprised of traditional didactic lectures, supplemented with an ECE program consisting of case based lectures and a hospital visit to see patients. A focus group discussion was conducted with the medical students and, based on the themes that emerged from it, a questionnaire was developed and administered to further enquire into the attitude of all the students towards ECE in learning endocrine physiology.

The students in their feedback commented that ECE increased their interest for the subject and motivated them to read more. They also felt that ECE enhanced their understanding of endocrine physiology, enabled them to remember the subject better, contributed to their knowledge of the subject and also helped them to integrate their knowledge. Many students said that ECE increased their sensitivity toward patient problems and needs. They expressed a desire and a need for ECE to be continued in teaching endocrine physiology for future groups of students and also be extended for teaching other systems as well. The majority of the students (96.4%) in their feedback gave an overall rating of the program as good to excellent on a 5 point Likert scale.

**Summary:**

The ECE program was introduced as an alternative approach to reinforce didactic instruction in endocrine physiology for the first year medical students. The study demonstrated that students clearly enjoyed the experience and perceived that it was valuable. This method could potentially be used for other basic science topics as well.

## Background

Teaching endocrine physiology to the first year medical students, in ways to make it interesting and also enhance their learning has always been a challenge. Student motivation and performance improve when the instruction is adapted to student learning preferences and styles [1]. Various methods are being used as alternative approaches to reinforce didactic instruction in endocrine physiology, such as case-stimulated learning [2], problem-based learning [3], patient-centred learning [4] and multiple-format sessions [5].

Early clinical exposure (ECE) involves an active, experiential learning from patients with practicing clinicians, designed to be the 'beginning of a life-time of learning focused on the patient' [6]. ECE programs are an increasingly widespread component of undergraduate medical education. There seems to be no "best" way to conduct ECE [7].

Breaking away from the practice of having only traditional didactic lectures, ECE program was also included in teaching endocrine physiology to the first year medical students of our institution. The attitude of the first year medical students toward ECE in learning endocrine physiology was assessed.

This paper describes the pros and cons of the various methodologies mentioned above as alternative approaches to reinforcing didactic instruction in endocrine physiology. It then goes on to describe the ECE program in learning endocrine physiology, and the student reactions to it as an alternative approach worthy of consideration.

## Discussion

### Case-stimulated learning

In this approach, each lecture began with the projection of one to three patient problems followed by several questions. This was followed by the traditional lecture, after which the patient problem(s) were projected again with the questions. Using the student responses, the instructor highlighted physiological concepts from the lecture [2].

This method provides a link between basic physiological concepts and clinical presentations. It also involves active learning. When there is time constraint, the questions are not discussed in the class and the students have to tackle it by themselves. In such scenarios, active learning may be lacking. Problem solving needs to be emphasized more in this method.

### Problem-based learning within lectures

This is not to be confused with the traditional problem based learning, which involves facilitated small group discussions conducted over several sessions, where the students form their own learning objectives. In this particular instance, the lecture began with the projection of one to three relevant cases with questions. After the traditional didactic lecture, the case was read in detail and the questions addressed to the students. The answers from the students were used to emphasise the content of the lecture [3].

An important aspect of this approach is the facilitated discussion at the end of the class. The instructor uses the answers of the students to further reinforce the lecture concepts. The disadvantage of this approach is that discussing the cases reduces time for sharing newer concepts in the field.

### Patient-centred learning curriculum

This approach is based on patient cases and is called patient-centred learning (PCL). On the first day of this curriculum, the students began to analyze the patient case and identify basic science learning issues for each member in the group to present in the next session two days later. After presentation and discussion of the learning issues, the faculty gave them a set of learning objectives, which the students used for further reading and presentation two days later. This was followed by presentation of the case by a physician. He also brought in a patient with the same disease for discussion and interaction [4].

The students were more receptive to this approach. Based on feedback from clinical students who had passed through this curriculum, they were better equipped to analyze clinical problems, find and apply appropriate basic science knowledge, and present their patients than students from the previous traditional curriculum. The limited objective performance data revealed no major gaps of basic science knowledge. The challenge faced in this particular methodology was increased faculty time for teaching [4].

### Multiple-format sessions for teaching endocrine physiology

In this approach, every session of three and a half hours duration was divided into four components: "a didactic lecture, a whole class discussion session, a quiz, and a patient presentation". The lecture was taken on the first day and the other components were done on the next day. It was done over two days so that the students could assimilate the lecture content before going on to the other three components [5].

This method allowed students to listen, read, discuss and solve problems in a large group format for every endocrine gland. This format was based on the key concept that repetition facilitates learning. This approach was appreciated by the students because of its variety. It allowed interactive learning. For the success of this approach it is necessary to have clinicians who like teaching and also are good teachers.

## Early Clinical Exposure Program (ECE)

The ECE program was used as a supplement to the traditional lectures in endocrine physiology. The program constituted of two case based lectures by an endocrinologist (clinician) and a hospital visit to see patients with the endocrinologist. The total number of medical students who completed all aspects of the study was 56, out of a class of 60 students.

The endocrinologist of Christian Medical College Hospital was informed 3 months in advance about the case based lectures. He was also requested to show patients at the hospital and to discuss their condition with the students.

After completion of the traditional didactic lectures on thyroid gland and pancreas, the endocrinologist gave the first case based lecture covering the same topics. The second case based lecture covering the adrenal, pituitary and parathyroid glands followed the remaining didactic lectures. This was followed by the hospital visit to see the patients.

### Case based lectures

**Figure 1:**
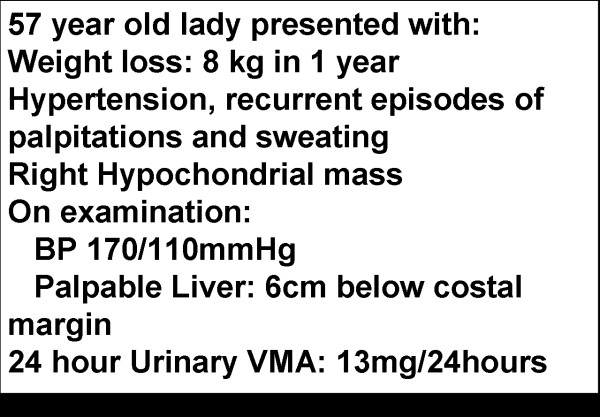
**Example of a case used during the Case Based Lectures**. Example of a case used during the Case Based Lectures; a patient with Pheochromocytoma.

The case based lectures were taken at the lecture hall with the help of an LCD projector. The lectures were based on patients who came to our hospital for treatment. Patient history, clinical examination findings, laboratory results (e.g. Figure 1), MRI scan images, X-ray images and photographs of patients were used in these lectures.

The first lecture was one hour in duration and the second lecture was for one and a half hours. The students were encouraged to ask questions and clarify any doubts.

### Hospital visit

The students were taken to the hospital to see the patients in the ward. They were divided into 3 groups of about 19 students per group. Each patient was discussed with the students by an endocrinologist, one group at a time. The discussion included patient history, symptoms, physiological basis of patient condition and treatment in brief. The clinical signs were demonstrated on the patients. The students were encouraged to ask questions and clear any doubts.

The patients that were seen were

1) A typical case of Acromegaly with large hands and feet, protruding lower jaw and other findings. This patient had a growth hormone secreting pituitary macroadenoma. He was waiting for a neurosurgery opinion as an outpatient and was requested to come to the ward.

2) A case of hyperthyroidism (Graves' disease) with exophthalmos and other findings. This patient had received radioactive iodine ablation in another hospital. He was admitted for initiation of pulse steroid therapy for severe Grave's opthalmopathy.

3) A typical case of hypothyroidism with myxedema, husky hoarse voice, sluggish reflexes, yellowish pigmentation of skin and other findings. This patient had Hoffman's syndrome with pseudohypertrophy of the calf muscles, Autoimmune Polyglandular Syndrome Type II (hypothyroidism and hypoadrenalism) and Vitamin B12 deficiency. He was admitted for initiating Eltroxine under coverage of Prednisolone to prevent an adrenal crisis and also for intramuscular Neurobion (Vitamin B12) injections for Megaloblastic anaemia.

4) A typical case of iatrogenic Cushing's syndrome with 'moon face', 'buffalo hump' and other findings. This patient was a renal allograft recipient who had features of renal rejection. He was admitted for a renal biopsy and additional immunotherapy, under Nephrology.

### Student feedback

Data was collected using focus group discussion and questionnaires. Approaches, recommended by Barzansky et al [8] were used, to explain the application of criteria of trustworthiness to the study methodology.

#### Focus group discussion

Seven students were chosen for a semi-structured focus group discussion on various aspects of the ECE program. The sample for the focus group discussion was a purposive sample, where the participant students were more articulate and representative of both genders with varying level of performance in class. Semi-structured questions were used to stimulate discussion. Based on the flow of the discussion the questions were modified and reframed keeping the goals of the study in mind. Three faculty members conducted the focus group discussion. One facilitated the discussion and the other two kept detailed notes of the discussion. The discussion was audio taped also. Later, the written and the audio-taped record were reviewed and transcribed in order to capture all the words and phrases.

Data analysis of students' focus group discussion was done using a grounded theory approach with constant comparative analysis [9,10]. Three of the authors (SS, ET and RV) analysed the comments of the focus group discussion and formulated themes independently. The themes that were identified were coded, and comments were assigned to the themes. The themes and the comments were discussed by them and 100% consensus was reached.

#### Questionnaire for the students

Based on the themes that emerged from the focus group discussion, a questionnaire was developed to further enquire into the attitude of all the students towards early clinical exposure. Out of a class of 60 students, 56 of them completed the questionnaire. The questionnaire had both open-ended questions, which yielded narrative comments, and structured questions, which yielded semi-quantitative data. A five point Likert scale, with a score of 1 = poor, 2 = not adequate, 3 = satisfactory, 4 = good and 5 = excellent, was used to find out the overall rating of the program by the students.

Quantitative data is reported in frequency distributions. Qualitative data analysis of the comments/phrases was done using an inductive approach. The themes were identified for each open ended question and comments assigned to each theme. Data was analyzed in the same way as for the focus group discussion. The themes and the comments were discussed by the authors and 100% consensus was reached.

We achieved data triangulation by using focus group discussion and a questionnaire. External audit, which involved review of the data by a colleague who was not a participant in the study, was done to ensure trustworthiness of the study. Member check, where the analysed data is reviewed by a participant in the study was done.

### Outcome

**Figure 2:**
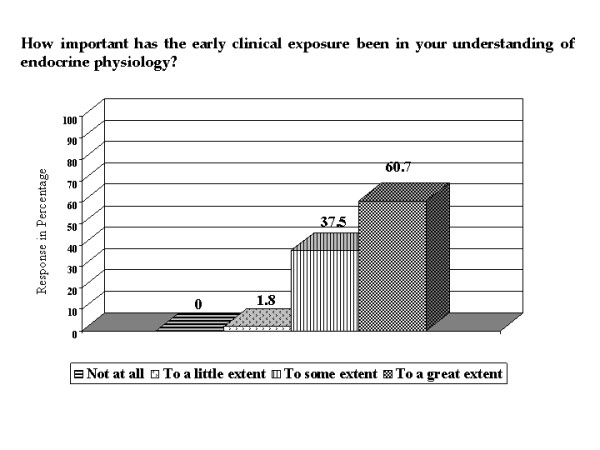
**Importance of ECE in understanding endocrine physiology**. Bar chart showing the extent to which ECE had helped the students in understanding endocrine physiology.

**Figure 3:**
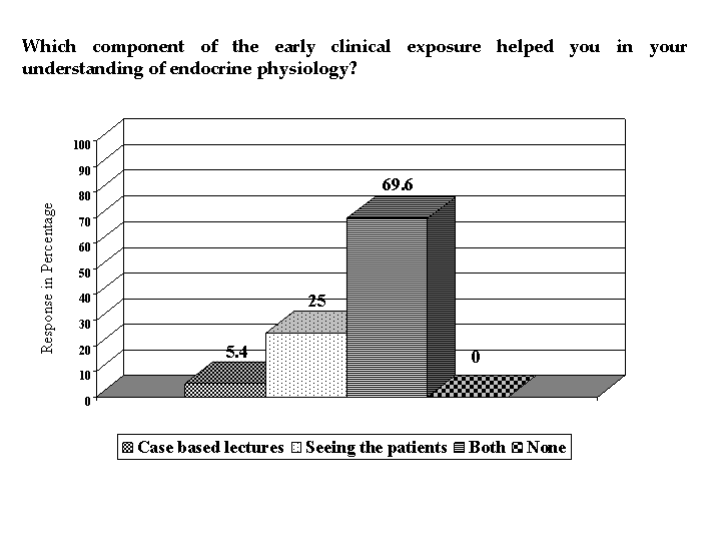
**Importance of the two components of ECE in understanding endocrine physiology**. Bar chart showing the extent to which the components of ECE helped the students in understanding endocrine physiology.

**Figure 4:**
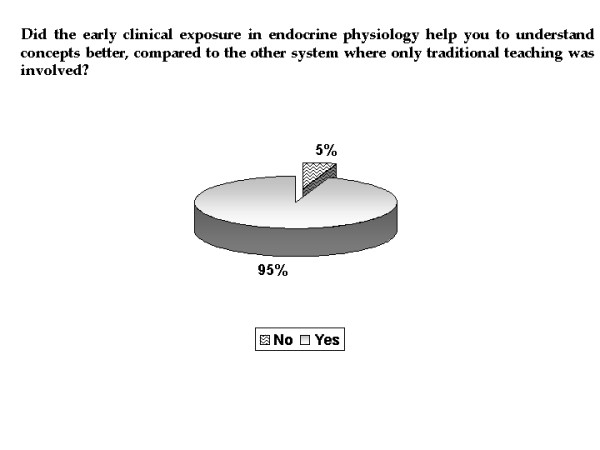
**Understanding concepts with ECE program, when compared to other systems taught only with didactic lectures**. Pie chart showing the effect of ECE in understanding endocrine physiology, as compared to other systems taught only by didactic lectures.

All the students commented that ECE helped them in their understanding of endocrine physiology and out of them, 60.7% felt that the help had been to 'great' extent (Figure 2). Commenting on what aspect of the ECE contributed to their understanding, 69.6% of students said that both case based lectures and seeing the patients helped (Figure 3). In comparison to the other systems where only didactic lectures were involved, 95% of the students felt that ECE in endocrine physiology helped them to understand the concepts better (Figure 4). Typical student comments are included in table 1.

**Table 1:** Typical student comments towards ECE in learning endocrine physiology

**Understanding of endocrine physiology:**	"*helped us develop a better understanding of the subject"* *"seeing the classical features in patients helped in understanding the subject deeper"* *"we could understand well instead of just memorizing causes and symptoms of disease"* *"made the concepts clear"*
**Acquiring knowledge:**	*" many things which we would have overlooked were brought to our notice in the case based lecture, normal values and especially treatment"* *"helped for the test"* *"the MRI scan showing the tumours in various locations re-enforced our knowledge"* *"seeing the patients make things stay much longer in your memory"*

**Integration of knowledge:**	*"we were able to relate what we saw with what we had read"* *"it was a nice class where we could integrate our knowledge"* *"clinical cases helped us to relate to the subject"* *"I understood how to apply physiology"*

**Development of interest:**	*"made theory come alive literally"* *"was interesting because it broke the monotony of 'by-hearting' symptoms and signs"* *"It is interesting to see the things we read in textbooks"* *"it really really did... made me feel like a doc"*

**Motivation to read:**	*"practical exposure to patients gave us more interest to go and read up that day"* *"seeing the patients motivated us to read more about what we didn't know"* *"I really held a great interest towards my textbook after the visit"* *"case based lectures were motivating and informative"*

**Development of sensitivity towards patient problems and needs:**	*"made us more sensitive toward them as we actually saw their plight"* *"helped me realise how much trouble each disease causes and be sensitive to the patients"* *"yes, the hospital visit helped....especially the patient with Cushing's syndrome"* *"helped to see how difficult these diseases are for the patient"*

**What they liked:**	"*It was something like a play-way-method of learning"* *"We learnt more about clinical applications than we otherwise would have"* *"seeing cases about which we can only read"* *"good case studies"* *"made learning interesting"* *"why we are studying physiology was clear"*

When asked if ECE had contributed toward acquiring knowledge in endocrine physiology, out of the 49 respondents to this question, 47 of them felt that it did contribute. Typical student comments are included in table 1. When asked in general what they liked about ECE, 10 students felt that ECE not only contributed towards acquiring knowledge but also helped to remember things better: *"seeing the patients in real life helped us remember the facts we studied" *and *"good picture memory"*.

One hundred percent of the students expressed that ECE helped them to integrate their knowledge of endocrine physiology. Typical student comments are included in table 1.

When asked if ECE had contributed towards developing interest in endocrine physiology, all 51 who responded said it did and to the question if ECE had motivated them to read endocrine physiology, all 51 who responded said it did. Typical student comments are included in table 1.

When asked if ECE had contributed toward developing sensitivity towards patient problems and needs, out of the 41 who responded, 38 of them said ECE had increased their sensitivity toward patient problems and needs. Typical student comments are included in table 1.

The students commented that the ECE program was *"very interesting" *and a *"good break from the usual theory classes"*. Typical student comments about what they liked about the program are included in table 1.

One hundred percent of the students said that the hospital visit to see patients increased their awareness of the disease conditions associated with endocrine physiology.

The majority (72.7%) of the students felt that ECE is essential in teaching endocrine physiology to future groups of students while the rest 27.3% felt it was advisable. None of them felt it was not needed. 52.7% of the students felt that it is essential to have ECE in teaching other systems while the remaining students said it was advisable. None of them said it was not needed.

In response to what they did not like about this ECE program, they said that the number of students per group while seeing the patient was large. When one group of students was seeing a patient, the other two groups had to wait. A few students felt that the number of patients seen was less. Some students felt that the second case based lecture was too long.

Suggestions for improvement of the programme included having smaller student groups for the hospital visit. Some students said *"Can probably have more patients"*. A few students felt that the waiting time could have been utilised in some other learning activity. Some students advocated an increase in frequency of hospital visits.

**Figure 5:**
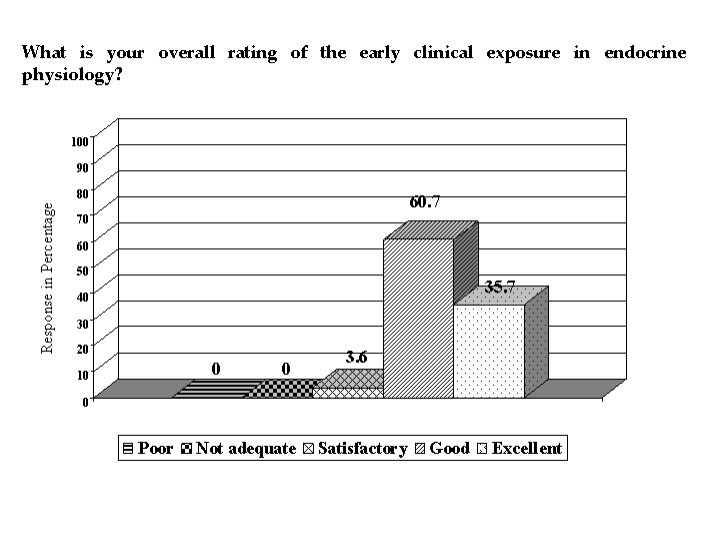
**Overall rating of ECE program**. Bar chart showing the overall rating of the ECE program by the students, on a five point Likert scale.

The students (96.4%) gave an overall rating of good/excellent to ECE (Figure 5) on a five point Likert scale. The mean score was 4.3, showing that the overall rating of ECE was good to excellent.

### Future direction

The apparent benefits of ECE include exposure to the health care system, instilling the qualities of a patient-centred humanistic physician and increasing motivation for classroom learning [7]. ECE forms a crucial part in the initiation of students into medicine [7]. During a time when students often spend long hours in the classroom, it serves to remind students why they want to be physicians [7]. Most students benefit from active learning strategies over the traditional lecture format [11].

In view of the good/excellent rating of the program by the students, ECE is planned to be implemented in teaching endocrine physiology for future groups of students, incorporating their suggestions. An objective analysis of the effectiveness of this approach could be made by comparing students' performance with and without the ECE program.

Christian Medical College, Vellore, India, is a 2234 bed multi-specialty, tertiary care teaching hospital. Patients are referred here from all over our country, which has a large population. Patients with endocrine disorders are admitted when necessary to the wards of our hospital for the treatment of the disease, for management of its complications or for surgical interventions either under the general surgery unit or neurosurgery unit. In teaching hospitals where many patients with endocrine disorders may not be admitted in the ward, video clipping of patients is a good option for similar teaching.

## Summary

Early clinical exposure was introduced as a teaching learning intervention in endocrine physiology for first year medical students. The study demonstrates that students clearly enjoyed the experience and perceived that it was valuable. The ECE program is an alternative approach to reinforce didactic instruction in endocrine physiology. This approach is adaptable to other medical physiology topics and to other basic science subjects as well.

## Abbreviations

ECE, Early Clinical Exposure

## Competing interests

The author(s) declare that they have no competing interests.

## Authors' contributions

SS, ET, RV and NT conceptualized the study. ET, RV and SS worked together to develop the research questions and the questionnaire. NN helped with the analysis. NT conducted the Case Based Lectures and the patient visit. ET, RV and SS conducted and analyzed the focus group discussion and questionnaire. All authors reviewed and modified a final interpretive summary. All authors read and approved the final manuscript.

## Pre-publication history

The pre-publication history for this paper can be accessed here:


